# α-Lipoic Acid Reduces Infarct Size and Preserves Cardiac Function in Rat Myocardial Ischemia/Reperfusion Injury through Activation of PI3K/Akt/Nrf2 Pathway

**DOI:** 10.1371/journal.pone.0058371

**Published:** 2013-03-07

**Authors:** Chao Deng, Zhongchan Sun, Guang Tong, Wei Yi, Li Ma, Bijun Zhao, Liang Cheng, Jinzhou Zhang, Feng Cao, Dinghua Yi

**Affiliations:** 1 Department of Cardiovascular Surgery, Xijing Hospital, Fourth Military Medical University, Xi’an, China; 2 Department of Cardiology, Xijing Hospital, Fourth Military Medical University, Xi’an, China; 3 Department of Anesthesia, Navy General Hospital, Beijing, China; University of Otago, New Zealand

## Abstract

**Background:**

The present study investigates the effects and mechanisms of α-Lipoic acid (LA) on myocardial infarct size, cardiac function and cardiomyocyte apoptosis in rat hearts subjected to in vivo myocardial ischemia/reperfusion (MI/R) injury.

**Methodology/Principal Findings:**

Male adult rats underwent 30 minutes of ischemia followed by 3, 24, or 72 h of reperfusion. Animals were pretreated with LA or vehicle before coronary artery ligation. The level of MI/R- induced LDH and CK release, infarct size, cardiomyocyte apoptosis and cardiac functional impairment were examined and compared. Western blot analysis was performed to elucidate the mechanism of LA pretreatment. The level of inflammatory cytokine TNF-α released to serum and accumulated in injured myocardium as well as neutrophil accumulation in injured myocardium were also examined after MI/R injury. Our results reveal that LA administration significantly reduced LDH and CK release, attenuated myocardial infarct size, decreased cardiomyocytes apoptosis, and partially preserved heart function. Western blot analysis showed that LA pretreatment up-regulated Akt phosphorylation and Nrf2 nuclear translocation while producing no impact on p38MAPK activation or nitric oxide (NO) production. LA pretreatment also increased expression of HO-1, a major target of Nrf2. LA treatment inhibited neutrophil accumulation and release of TNF-α. Moreover, PI3K inhibition abolished the beneficial effects of LA.

**Conclusions/Significance:**

This study indicates that LA attenuates cardiac dysfunction by reducing cardiomyoctyes necrosis, apoptosis and inflammation after MI/R. LA exerts its action by activating the PI3K/Akt pathway as well as subsequent Nrf2 nuclear translocation and induction of cytoprotective genes such as HO-1.

## Introduction

Restoring coronary blood flow (reperfusion) using pharmacological or mechanical interventions following acute myocardial ischemia is essential for the salvation of viable myocardium. Paradoxically, reperfusion itself causes cell damage and cell death mostly by initiating a localized oxidative burst and regional inflammatory response, referred to as “reperfusion injury” [1]. Myocardial reperfusion injury, which is defined as myocardial injury caused by restoration of coronary blood flow after an ischemic episode, culminates in the death of cardiomyoctyes that were viable immediately before reperfusion [2]. In animal studies, reperfusion injury is suggested to be responsible for up to 50% of the final infarct size. A number of strategies and pharmacological agents are shown to ameliorate reperfusion injury in these animal models. However, translation of these strategies and agents to the clinical setting has been disappointing [3]. To improve clinical outcomes in acute myocardial infarction, it is of pivotal importance to develop new pharmacological agents for limiting reperfusion injury and preserving heart function.

α-Lipoic acid (LA) is a thiol antioxidant which can be found in food such as broccoli, spinach, and tomatoes. LA is recognized as a complement of α-keto acid dehydrogenase complexes of mitochondria and therefore plays a fundamental role in metabolism [4]. LA and its reduced form, dihydrolipoic acid (DHLA) are considered ideal antioxidants because they have a low redox potential so they not only directly scavenge ROS but also regenerate endogenous antioxidants such as glutathione and vitamins E and C [5]. LA also has diverse biodistribution, it is both water and lipid soluble and is widely distributed in cellular membranes, cytosol and extracellular spaces. LA is clinically approved and widely used for the treatment of diabetic polyneuropathy [6]. LA has also been described as a therapeutic agent for a number of conditions related to cardiovascular disease, including lipid abnormality [7], diabetes [8] and stroke [9]. In animal studies, LA has been shown to reduce cell damage and oxidative stress in organs subjected to ischemia/reperfusion (I/R) [10], [11]. The protection of LA is largely attributed to its antioxidant property, however, LA also exhibits distinct regulatory action on signal transduction processes involved in tissue damage and protection [12]. Multiple cell signaling pathways including PI3K/Akt/Nrf2 [13], p38MAPK [14], and nitric oxide (NO) singaling [15] have been shown to be activated by LA and mediate its pharmacological effects. Of note, these pathways also play pivotal roles in cardiomyocyte survival after I/R injury. However, the role of these signaling pathways in LA-mediated protection against MI/R is unclear. In the present study, we investigated 1) whether LA protects rat myocardium from I/R injury in vivo and 2) the possible role of PI3K/Akt/Nrf2, p38MAPK, and nitric oxide (NO) in LA protection against MI/R.

## Methods

### Animals

The experiments were performed in adherence with the National Institutes of Health Guidelines on the Use of Laboratory Animals and were approved by the Fourth Military Medical University Ethic Committee on Animal Care (Approval ID: 2009055). Adult male Sprague-Dawley (SD) rats, weight 220 to 250 g were purchased from the animal center of the Fourth Military Medical University. A total number of 120 rats were used in this study. Sodium pentobarbital (Sigma, 40 mg/kg, IP ) was used for anesthesia**.** In the first phase of the study, different dosages of LA were administered to determine the optimal dosage for protection. Thirty rats were randomly divided into 6 groups and given saline or 5,10,15,25 or 50 mg/kg of LA before MI/R. The extent of protection was determined by infarct size reduction. After determining 15 mg/kg as the optimal dosage, rats were randomly divided into the following groups (n = 10): (1) sham operation group (sham I/R); (2) I/R+vehicle group (I/R+ V); (3) I/R+ α-Lipoic acid group (I/R+LA); and (4) α-Lipoic acid +I/R+wortmannin group (I/R+LA+W). LA (99%, Sigma, USA, 15 mg/kg) was given by tail vein injection 30 min before ischemia. Wortmannin (Sigma, USA, 15 µg/kg) was injected via tail vein 5 min before LA injection. In the latter phase of the study, in order to verify the role of PI3K/Akt pathway, LY294002 (0.3 mg/kg, IV), another PI3K/Akt specific inhibitor, was administered 5 min before LA injection.

### LDH and CK Release Evaluation

After 3 h of reperfusion, blood samples were collected from the right ventricle and centrifuged at 3000 g for 10 min to isolate serum. Myocardial cellular damage was evaluated by measuring lactate dehydrogenase (LDH) and creatinine kinase (CK) activity in plasma using commercially available assay kits (Jianchen, Nanjing, China).

### Construction of MI/R Animal Model

The MI/R animal model was constructed by left anterior descending coronary artery (LAD) ligation. In brief, rats were anesthetized with sodium pentobarbital (Sigma, 40 mg/kg, IP ). After intubation, the chest was opened through a left thoracic incision to expose the heart. A 6–0 silk suture slipknot was placed around the LAD. After 30 min of ischemia, the slipknot was released, allowing the myocardium to be reperfused. Sham-operated rats underwent the same surgical procedure except that the suture placed around LAD was not tied.

### Measurement of Myocardial Infarct Size

Myocardial infarct size was evaluated by Evans Blue/2,3,5-triphenyl-2H-tetrazolium chloride (TTC) staining as previously described [16]. TTC is enzymatically reduced to red 1,3,5-triphenylformazan in viable tissues due to the activity of various dehydrogenases. Hearts were reperfused for 24 h to clear dehydrogenases from necrotic tissue. After 24 hours of reperfusion, the LAD was religated, and 1 ml of 2% Evans Blue dye was injected into aorta. The heart was quickly excised, frozen at −20°C before being cut transversally into 1 mm thick slices, and incubated in 1% TTC (Sigma, USA) at 37°C for 10 min. The Evans Blue-stained blue area is the area not at risk (ANAR). The area stained red by TTC represents ischemic but viable tissue. Infarcted myocardium was not stained by either TTC or Evans Blue and is more pale than TTC stained area. The area of infarct size (IS) and area at risk (AAR) were measured digitally using Image Pro Plus software (Media Cybernetics). IS and AAR were expressed as percentages of the left ventricular area (IS/LV and AAR/LV respectively).

### Determination of Myocardial Apoptosis

Myocardial apoptosis was determined by terminal deoxynucleotidyl transferase-mediated dUTP-biotin nick end labeling (TUNEL) staining and caspase-3 activity after 3 h of reperfusion. TUNEL staining was performed with fluoresceindUTP (In Situ Cell Death Detection Kit; Roche Diagnostics) for apoptotic cell nuclei and 4′,6-diamidino-2-phenylindole (DAPI) (Sigma, USA) for all cell nuclei as previously described [17]. The apoptotic index (AI) was determined as the number of TUNEL-positive nuclei divided by the total number of nuclei stained with DAPI from a total of 40 fields per heart (n = 5). Cardiac caspase-3 activity was determined using a caspase-3 colorimetric assay kit (Chemicon, Temecula, CA) following the manufacturer’s instructions. Heart tissue samples for determination of myocardial caspase-3 activity were obtained from the margin of infarct (peri-infarct) areas. Cleaved caspase-3 levels were examined with western blot.

### Determination of Cardiac Function

To avoid interference of acoustic signal by residual air trapped inside the chest cavity, echocardiography was conducted after 72 h of reperfusion, by which time most of the residual air has been absorbed. Baseline echocardiography was obtained 30 min before ischemia. Rats were sedated with 3% isoflurane inhalation. Cardiac dimensions and function were studied by M-mode echocardiography using an echocardiography system with a 15-MHz linear transducer (VisualSonics Vevo 2100, Canada). Left ventricular end-diastolic diameter (LVEDD) and left ventricular end-systolic diameter (LVESD) were measured on the parasternal left ventricular long axis view. All measurements represent the mean of 5 consecutive cardiac cycles. Left ventricular end-systolic volume (LVESV), left ventricular end-diastolic volume (LVEDV) and left ventricular ejection fraction (LVEF) were calculated by computer algorithms. All of these measurements were performed in a blinded manner.

### Western Blot Evaluation

Western blot samples were extracted from the myocardium after 3 h or 24 h of reperfusion. For detection of changes in kinase phosphorylation, a fast, transient post-translational modification that can be dramatically changed in the acute phase of reperfusion, samples were extracted after 3 h of reperfusion. Samples extracted after 24 h of reperfusion were used for detection of proteins that were induced by MI/R, such as iNOS and HO-1. Nuclear and cytoplasm protein are isolated using an isolation kit (pierce, USA) according to the manufacturer’s instructions. In another set of samples, whole heart protein was extracted from total homogenous of heart tissue. In brief, heart tissue was first groond to power in liquid nitrogen and then lysed in ice-cold RIPA buffer containing 1% protease inhibitor cocktail (Sigma, USA). The homogenate was then incubated on ice for 30 min before centrifugation at 12,000 g for 20 min at 4°C. The supernatant was transferred, aliquoted and stored at −80°C. After protein concentration measurement with the modified Bradford assay (Bio-Rad Laboratories, Hercules, USA), proteins were separated by electrophoresis on SDS-PAGE and transferred to nitrocellulose membranes and probed with primary antibodies against HO-1 (1∶500), Nrf2 (1∶500), iNOS (1∶200Santa Cruz, USA), Akt (1∶1000), phospho-Akt (Ser-473) (1∶1000), p38-MAPK (1∶1000), phospho-p38-MAPK (1∶1000), eNOS (1∶1000), phospho-eNOS (1∶1000, Cell Signaling, USA), overnight at 4°C followed by incubation with the corresponding secondary antibodies at room temperature for 1 h. The blots were visualized with ECL-Plus reagent (GE Healthcare, USA).

### Determination of Tissue Myeloperoxidase (MPO) and Tumor Necrosis Factor-alpha (TNF-α) Level

After 3 h of reperfusion, myocardial samples were taken from the AAR zones for MPO activity analysis and TNF-α level measurement. The activity of MPO was measured using a commercially-available kit (Jianchen, Nanjing, China) and expressed as units per 100 mg of tissue. Myocardial and serum contents of TNF-α were measured using enzyme-linked immunosorbent assay kits (R&D Systems, USA) according to the manufacturer’s instructions. Blood samples were draw from the right ventricle and centrifuged at 3000 g for 10 min to isolate serum samples. The protein concentration of samples was measured using the Bio-Rad Protein Assay (Bio-Rad Laboratories, Hercules, CA) and bovine serum albumin was used as the standard.

### Measurement of NOx Content in Myocardium

The myocardial NOx concentration was measured as previously described [18]. In brief, after 3 h or 24 h of reperfusion, myocardial samples from the AAR were rinsed and homogenized in deionized water (1∶10, wt/vol) before centrifugation at 3,000 g for 5 min. NOx concentrations in the supernatant were quantified using a NO detection kit (Jianchen, Nanjing, China) following the manufacturer’s instruction.

### Statistical Analysis

One-way ANOVA was conducted across all groups first followed by a Bonferroni post-hoc correction between all group comparisons. Data are expressed as mean±SEM. Significance was accepted at *p*<0.05. GraphPad Prism-5 statistical software (La Jolla, CA) was used for data analysis.

## Results

### 15 mg/kg of LA Produces Potent Protection

15, 25 or 50 mg of LA all significantly reduced infarct size compared to control. However, 5 mg or 10 mg of LA did not decrease infarct size compared to control. Additionally, 25 mg or 50 mg of LA did not enhance protection beyond that of 15 mg (Figure S1a,b). We chose 15 mg/kg as the dosage for the rest of our study.

### LA Reduces CK and LDH Release After I/R Injury

**Figure 1:**
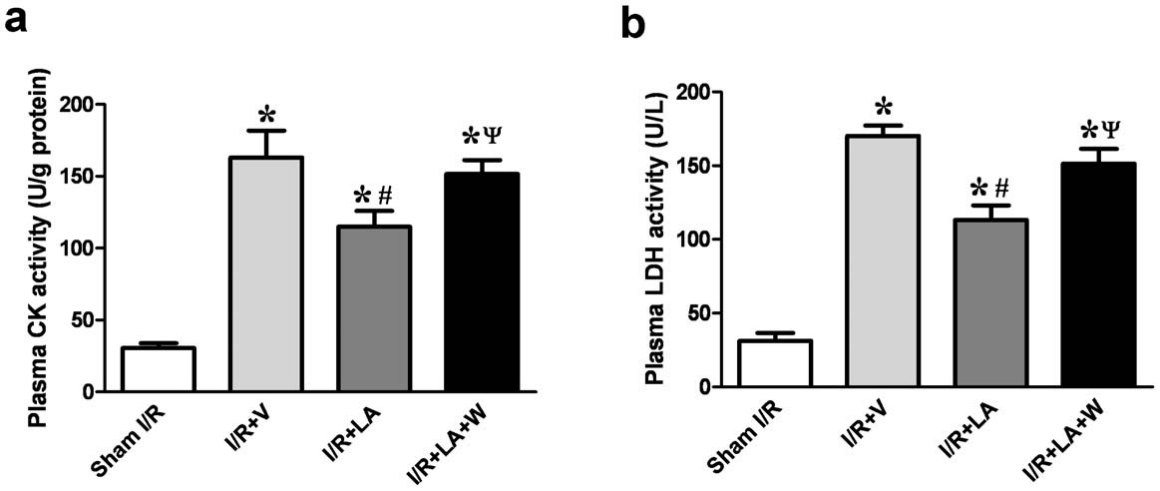
LA reduces LDH and CK release. LA reduced CK and LDH level after I/R injury in rat serum. PI3K inhibitor wortmannin cotreatment statistically increased the release of LDH and CK compared with LA treated alone. The columns and errors bars represent means and SEM. *p<0.05 vs sham I/R, ^#^ *p*<0.05 vs I/R+V, ^ψ^ *P*<0.05 vs I/R+LA.

LA significantly decreased the serum levels of the necrotic cell death markers, lactate dehydrogenase (LDH) and creatinine kinase (CK), compared with the I/R+V group (CK: 114.9±11.07 vs 163.1±7.648 U/g protein, *P* = 0.005; LDH: 113.3±9.775 vs 170.2±7.087 U/L, *P* = 0.0015). However, this effect was largely alleviated by the PI3K inhibitor wortmannin (CK: 151.6±9.623 vs 114.9±11.07 U/g protein, *P* = 0.031; LDH: 151.5±9.962 vs 113.3±9.775 U/L, *P = *0.026) (Figure 1a,b). This reduction of cardiac enzyme release by LA was also abolished by LY294002, another specific PI3K inhibitor (Figure S3a,b).

### Decreases Infarct Size and Cardiomyocytes Apoptosis After I/R Injury

**Figure 2:**
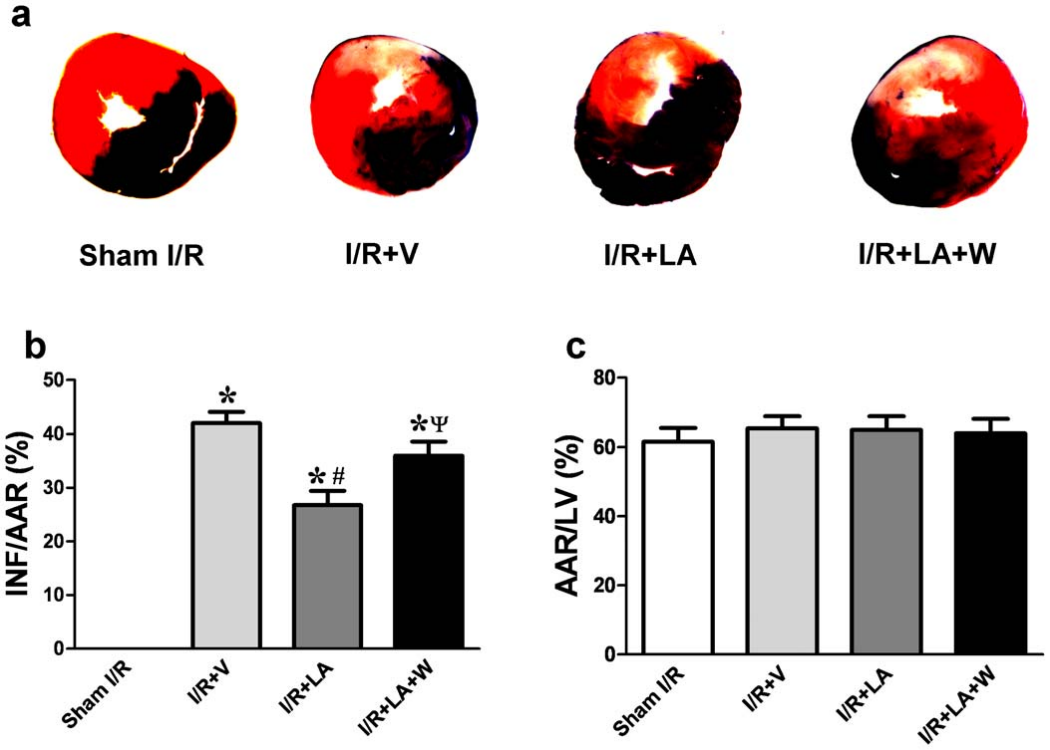
LA decreases infarct size in rats subjected to I/R injury. Representative illustrations of infarct size as stained by Evans Blue and TTC (a). LA decreased infarct size compared with I/R+V group and wortmannin pretreated group (b). The area at risk showed no statistical difference between groups (c). The columns and errors bars represent means and SEM. *p<0.05 vs sham I/R, ^#^ *p*<0.05 vs I/R+V, ^ψ^ *P*<0.05 vs I/R+LA.

As shown in Figure 2, LA administration significantly decreased infarct size (26.77±2.653 vs 42.06±2.101, *P = *0.002) after 24 h of reperfusion compared with the I/R+V group. This effect was abolished by wortmannin treatment (35.97±2.65 in wortmannin group vs 26.77±2.653 in LA group, *P* = 0.002) (Figure 2a,b). No significant difference in the size of the AAR was found between groups (Figure 2c). This reduction of infarct size by LA was also abolished by LY294002 (Figure S3c).

**Figure 3:**
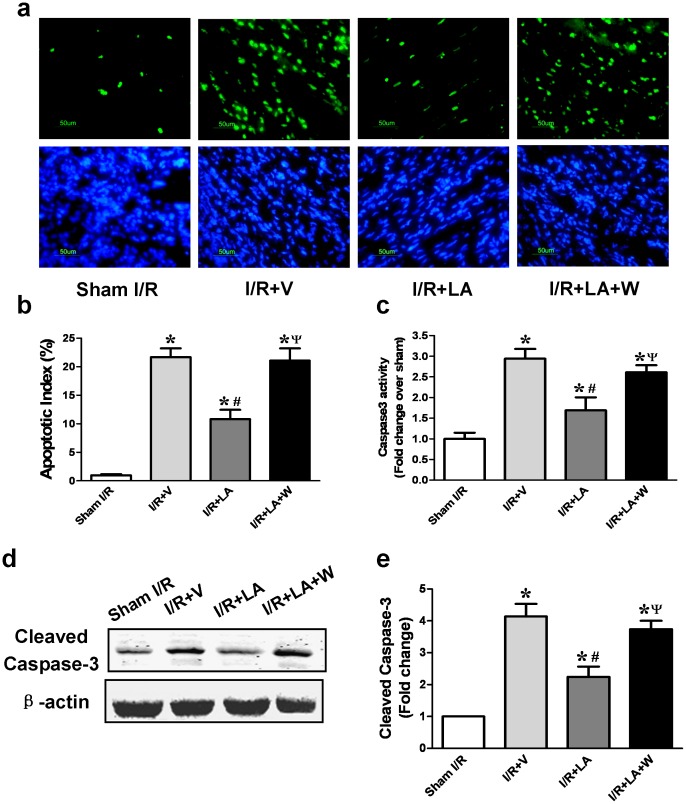
LA decreases cardiomyocyte apoptosis in rats subjected to MI/R injury. Representative photomicrograph showed that TUNEL-positive cardiomyocytes were more frequently observed in I/R+V group and the wortmannin-pretreated group compared with the LA group (a). The number of TUNEL-positive cardiomyocytes (in green, DAPI in blue) was significantly reduced in the LA group compared to the I/R+V group and the wortmannin pretreated group (b). LA treatment also significantly decreased caspase-3 activity compared with I/R+V group and wortmannin-pretreated group (c). LA decreased cleaved caspase-3 expression as compared with the I/R+V group and the wortmannin-pretreated group (d, e). The columns and errors bars represent means and SEM. *p<0.05 vs sham I/R, ^#^ *p*<0.05 vs I/R+V, ^ψ^ *P*<0.05 vs I/R+LA.

Cardiomyocytes apoptosis was evaluated by TUNEL/DAPI dual staining, caspase-3 activity and cleaved caspase-3 level. Quantitative analyses demonstrated that the percentage of TUNEL-positive cardiomyocytes was significantly lower in the LA group than in the I/R+V group (10.8±1.64 vs 21.68±1.548, *P* = 0.0013). However, this effect was largely alleviated by wortmannin (21.08±2.139 vs 10.8±1.64, *P = *0.0051) (Figure 3a, b). Compared with the vehicle treated I/R group, LA-treated hearts had suppressed caspase-3 activity (1.689±0.3167 vs 2.942±0.2367, *P* = 0.013), indicating lower pro-apoptotic enzyme activity in this group. This effect was abolished by wortmannin (2.612±0.1706 vs 1.689±0.3167, *P* = 0.033) (Figure 3c). Western blot analysis also demonstrated lower cleaved caspase-3 levels, in the LA group as compared with both the I/R+V group and the wortmannin group (Figure 3d, 3e). This reduction of cardiomyoctye apoptosis by LA was also abolished by LY294002 (Figure S3d,e).

### LA Partially Preserved Left Ventricular Function After I/R Injury

**Figure 4:**
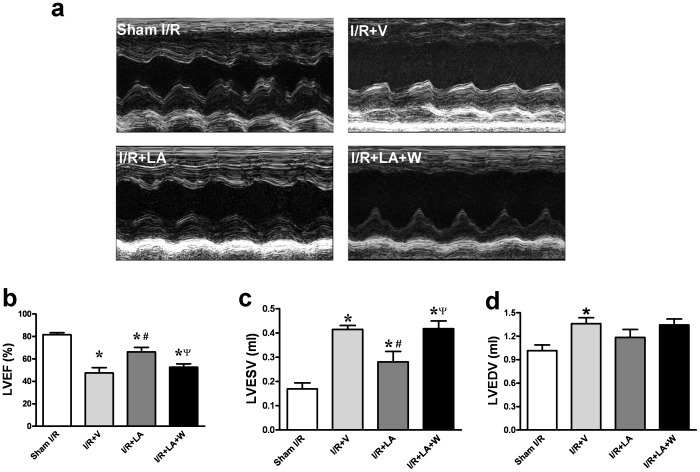
LA partially preserved left ventricular function evaluated by echocardiograpy. Representative image of echocardiography showed that the LA group has better left ventricular free wall motion compared with the I/R+V group and the wortmannin-pretreated group (a). LA administration significantly enhanced LVEF as compared with the I/R+V group and the wortmannin-pretreated group (b). LA significantly inhibited the increase of LVESV compared with the I/R+V group and the wortmannin pretreated group (c,d). LVEF: Left ventricular ejection fraction; LVESV: Left ventricular end-systolic volume; LVEDV: Left ventricular end-diastolic volume. The columns and errors bars represent means and SEM. *p<0.05 vs sham I/R, ^#^ *p*<0.05 vs I/R+V, ^ψ^ *P*<0.05 vs I/R+LA.

Left ventricular function was evaluated with echocardiography after 30 min ischemia and 72 h of reperfusion. No significant difference in baseline echocardiography was observed between groups (Figure S2a,b,c). Compared to the sham I/R group, I/R injury caused a significant decrease of LVEF (81.53±1.747 vs 47.42±4.775, *P*<0.001) and a dramatic increase of LVESV (0.1694±0.0247 vs 0.4148±0.0166, *P*<0.001) and LVEDV (1.015±0.0735 vs 1.359±0.0755, *P* = 0.011). LA administration significantly blunted the reduction of LVEF caused by I/R injury (66.22±3.989 vs 47.42±4.775, *P* = 0.017) while wortmannin alleviated this effect (52.58±3.064 vs 66.22±3.989, *P* = 0.027) (Figure 4a, 4b). LA significantly inhibited the increase of LVESV compared with the I/R+V group (0.28±0.044 vs 0.4148±0.017 ml, *P* = 0.021) and the wortmannin group (0.28±0.044 vs 0.417±0.032 ml, P = 0.036) (Figure 4c). The LA group showed a trend toward LVEDV decrease compared with both the I/R+V group (1.183±0.103 vs 1.359±0.075 ml, P = 0.206) and the wortmannin-treated group (1.183±0.103 vs 1.342±0.076 ml, P = 0.251) (Figure 4d), however, no statistical significance was observed.

### LA Treatment Inhibits Neutrophil Infiltration and TNF-α Level After I/R Injury

**Figure 5:**
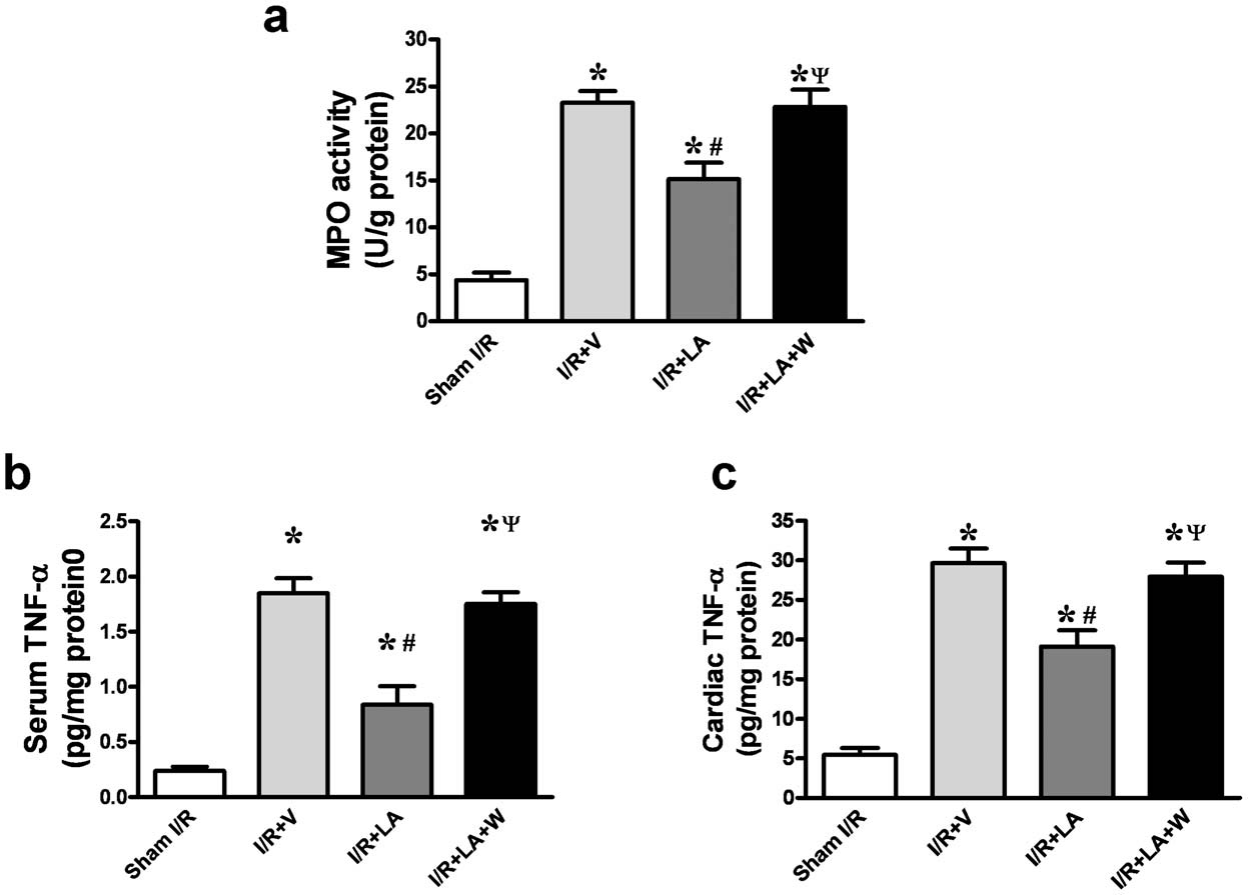
LA alleviates neutrophil infiltration and TNF-α levels elevation. LA reduced the MPO activity in I/R compared with vechicle and the wortmannin group (a). LA reduced serum levels of TNF-α compared with the I/R+V group and the wortmannin-pretreated group (b). LA reduced cardiac levels of TNF-α compared with the I/R+V group and the wortmannin-pretreated group (c). The columns and errors bars represent means and SEM. *p<0.05 vs sham I/R, ^#^ *p*<0.05 vs I/R+V, ^ψ^ *P*<0.05 vs I/R+LA.

After 3 h of reperfusion, the activity of MPO was significantly increased in the I/R+V group compared to the sham I/R group (23.3±1.211 vs 4.389±0.786 U/100 mg, *P*<0.001). LA reduced MPO activity in the I/R group compared with the I/R+V group (15.15±1.738 vs 23.3±1.211 U/100 mg, *P* = 0.0085) and the wortmannin group (15.15±1.738 vs 22.83±1.829 U/100 mg, *P* = 0.023) (Figure 5a), indicating a decrease of neutrophil accumulation in LA treated heart. ELISA analysis demonstrated that I/R resulted in a dramatic increase of TNF-α in both serum (1.849±0.135 vs 0.239±0.0368 pg/mg protein, *P*<0.001) and the injured myocardium (29.64±1.851 vs 5.453±0.854 pg/mg protein, *P*<0.001) compared with sham group (Figure 5b,5c). LA inhibited the increase of TNF-α in both serum (1.849±0.135 vs 0.839±0.167 pg/mg protein, *P* = 0.0033) and the myocardium (29.64±1.851 vs 19.11±2.044 pg/mg protein, *P* = 0.0088). Reductions of TNF-α in both serum and the myocardium were abolished by wortmannin administration (serum: 0.839±0.167 vs 1.751±0.106 pg/mg protein, *P* = 0.0037; myocardium: 19.11±2.044 vs 27.93±1.782 pg/mg protein, *P* = 0.017) (Figure 5b,5c).

### LA does not Affect Activation of p38 MAPK

**Figure 6:**
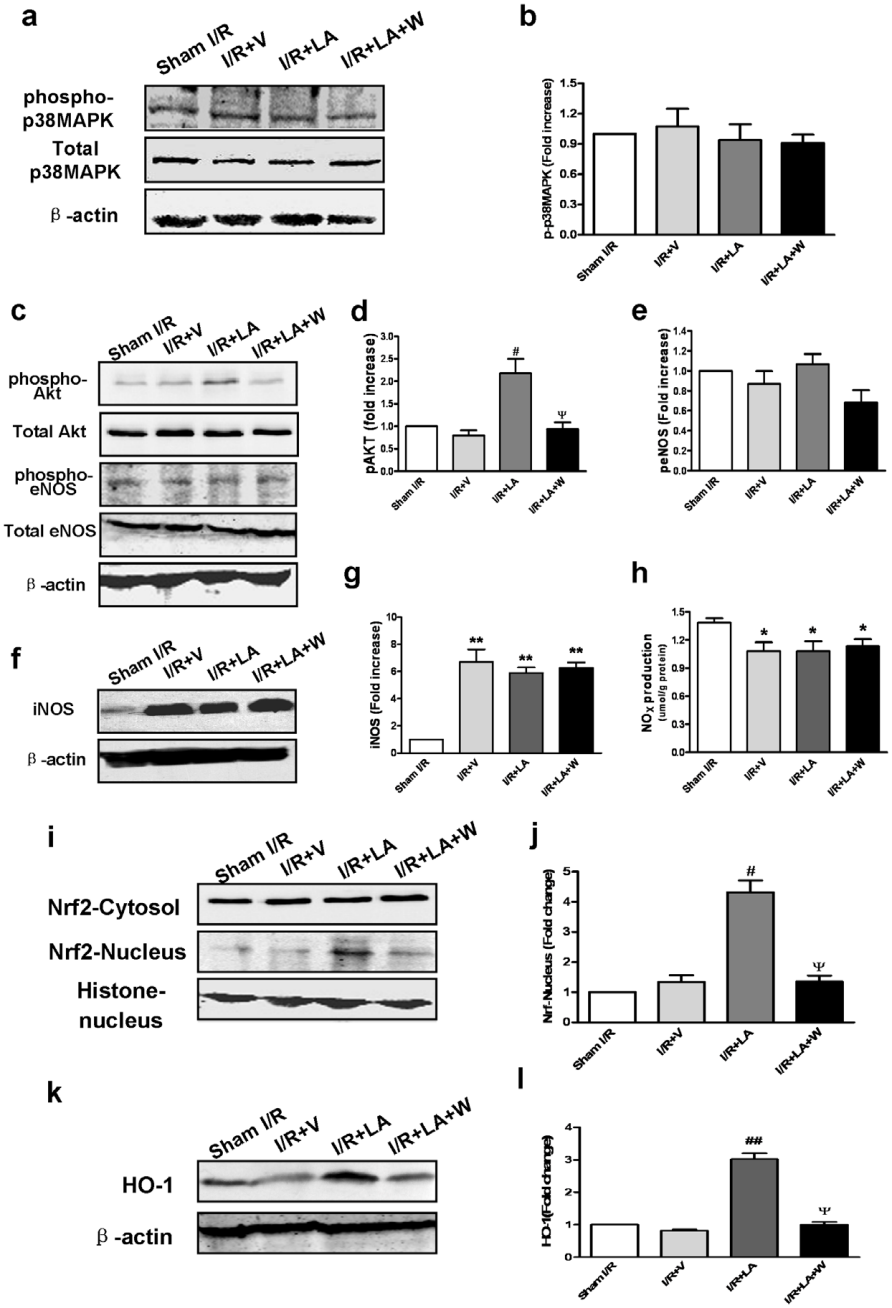
Western blot analysis. Western blot analysis showed no difference of phosphorylated p38MAPK level between the LA and the I/R group (a,b). LA treatment increased the level of phosphorylated Akt as compared with the I/R+V group and the wortmannin pretreated group (c, d). No difference of phosphorylated eNOS or NOx level was observed between the LA and the I/R group after 3 h of reperfusion(e, f). After 24 h of reperfusion, no difference of iNOS or NOx level between the LA and the I/R group was observed(g, h). LA enhanced nuclear Nrf2 protein level but did not significantly change the level of Nrf2 in the cytoplasm. Wortmannin abolished the effect of LA on nuclear Nrf2 level (i, j). LA increased HO-1 gene transcription and expression, detected by Real Time PCR and western blot respectively, compared to the I/R+V and I/R+LA+W groups(k,l). The columns and errors bars represent means and SEM. *p<0.05 vs sham I/R, ^#^ *p*<0.05 vs I/R+V, ^ψ^ *P*<0.05 vs I/R+LA.

Due to a previous report that LA exerts cardioprotection by modulating JNK1/2 and ERK1/2, two members of MAPK family [19], we examined the impact of LA on phosphorylation of p38MAPK, the other important member of MAPK family. However, pretreatment with LA exerted no significant effect on phosphorylation of p38 MAPK (Figure 6a, 6b) in rat myocardium subjected to 30 min of ischemia and 3 h of reperfusion. LA preconditioning appears not to activate p38 MAPK, and therefore p38MAPK is less likely to play important role in LA induced cardioprotection in this model of rat myocardial I/R injury.

### Increases Phosphorylation of Akt

The PI3K/Akt pathway was a potential cell signaling pathway mediating the protective effect of LA [20]. Phosphorylation of Akt was examined by western blot. Compared to the I/R+V group, LA significantly increased phosphorylation of Akt. This increase of Akt phosphorylation was abolished by co-administration of wortmannin (Figure 6c, 6d). Cotreatment with LY294002 also blocked the LA-induced activation of Akt (Figure S3f).

### LA has No Effect on Nitric Oxide Production

Among potential cytoprotective downstream targets of Akt, phosphorylation of eNOS and the subsequent production of NO have been shown to play a pivotal role in Akt-mediated protection. We examined whether LA increased eNOS phorsphorylation and NO production in the acute phase of reperfusion. After 3 h of reperfusion, eNOS phosphorylation and NO production were both reduced by MI/R, however, LA produced no effect on either eNOS phosphorylation(Figure 6c,e) or NO production(Figure 6h). LA is known to generate protection by preventing the induction of iNOS gene expression [21], [22], which can generate toxic amounts of NO in the late phase of reperfusion injury. After 24 h of reperfusion, NOx level were significantly elevated by MI/R injury, probably by induction of iNOS [23], however, LA did not produce any effect on the elevation of NOx or iNOS (Figure 6f, 6g, 6i). These results suggest that NO is unlikely to mediate LA-induced protection.

### LA Increases Nrf2 Nuclear Translocation

It is known that the redox-sensitive transcription factor 2 (Nrf2) plays an important role in cell survival following I/R. LA has been shown to induce Nrf2 nuclear translocation and activation. Nrf2 nuclear translocation in LA treated hearts was examined by nuclear protein extraction followed by western blot. Nrf2 levels in cardiomyocyte cytoplasm and nucleus were examined by western blot following nuclear and cytoplasmic protein isolation. Compared with the I/R+V group, the LA group showed no significant change in Nrf2 levels in cytoplasmic protein. In contrast, the Nrf2 levels in nuclear protein from the LA group was significantly elevated, indicating an increase of Nrf2 nuclear translocation (Figure 6i, 6j). Interestingly, this increase of Nrf2 nuclear translocation was completely abolished by Wortmannin. These observations indicate that LA promotes Nrf2 nuclear translocation at least partially through activating PI3K/Akt pathway.

### LA Increases HO-1 Gene Transcription and Expression

Having demonstrated that LA increased Nrf2 nuclear translocation, we set to identify the major target gene of Nrf2. Nrf2 nuclear translocation can upregulate expression of several antioxidative enzymes, such as HO-1, glutathione S-transferase, superoxide dismutase and NADPH-regenerating enzymes, all of which are recognized to play important role in combating oxidative stress. However, HO-1 promoter is known to have a larger number of antioxidant response elements (ARE) sequences that Nrf2 can bind to and induce its expression in a preferential manner [24]. In addition, increase of HO-1 expression has been shown to make the heart more resilient to I/R injury [25]. Therefore, we hypothesized that LA induced Nrf2 nuclear translocation increases HO-1 gene expression. After 24 h of reperfusion. HO-1 protein levels were significantly increased in the myocardium. This effect was blocked by co-administration of wortmannin(Figure 6k, 6l). These observations indicated that LA promotes HO-1 gene transcription and expression in myocardium subjected to MI/R. This effect is probably secondary to Nrf2 nuclear translocation caused by PI3K/Akt pathway activation.

## Discussion

In the present study, using an in vivo rat model of MI/R, we evaluated the protective effect of LA against MI/R and explored the mechanisms involved. Our results show that MI/R leads to necrosis and apoptosis of cardiomyocytes, myocardium inflammation and eventually myocardial infarct and heart function loss. Pretreatment with 15 mg/kg of LA significantly attenuated I/R-induced cardiomyocyte death, inflammation and most importantly, infarct size and cardiac dysfunction (decrease in LVEF, elevation in LVESV) concomitantly with an increase in Akt phosphorylation, Nrf2 nuclear translocation and HO-1 expression. All these effects were abolished by PI3K inhibition. Unexpectedly, LA did not influence p38MAPK activation or NO production. To the best of our knowledge, this is the first study demonstrating that LA pretreatment in vivo partially preserves heart function by reducing cardiomyocyte death and inflammation secondary to MI/R, and this protection is mediated, at least in part, by PI3K/Akt activation and subsequent Nrf2 nuclear translocation.

LA can be derived from almost all foods or synthesized by human organs like the liver. LA functions as a cofactor for multiple metabolic enzymes, such as pyruvate dehydrogenase and α-keto-glutarate dehydrogenase [26], [27]. Due to its property as a safe, powerful antioxidant, LA is widely used and well tolerated in the treatment of diabetic polyneuropathy [6]. However, the beneficial effects of LA seem to be only partially derived from its antioxidant properties. Since the finding of Konrad et al [28] first suggested that LA increases glucose uptake by specifically activating p38MAPK and GLUT4, abundant evidence has connected LA with cell signaling modulation [12].

Due to its antioxidant properties, LA has potential implications in treating oxidative injuries [12]; however, the mechanism of LA protection was poorly understood. Recently, LA has been reported to reduce myocardial I/R injury in vitro [29]. Using an in vivo model, we showed that pre-ischemic treatment with LA reduced infarct size, decreased cardiomyoctyes apoptosis, and most importantly, partially preserved heart function in rat myocardial I/R.

LA also reduced myocardial I/R induced inflammation, which could also contribute to its protective effect against I/R. Inflammatory processes including neutrophil infiltration and proinflammatory cytokines production, play a major role in the extension of myocardial damage after I/R, while anti-inflammatory therapies reduce myocardial reperfusion injury [30]. LA has been well documented to inhibit inflammation in multiple disease processes [31], [32], [33]. We therefore hypothesize that LA inhibits inflammation caused by I/R and this anti-inflammatory effect contributes to the protection against I/R. Our results from the present study show that in an in vivo model of myocardial I/R, LA pretreatment significantly reduces both serum and cardiac TNF-α production and blocks neutrophil accumulation in I/R myocardium, both of which are key elements of post-I/R inflammation.

To clarify the mechanism of LA preconditioning, we first investigated the activation of p38 MAPK. LA was shown to exert its protective effect against heart I/R by activating ERK1/2 and inhibiting JNK1/2, both of which are members of the MAPK family [19]. In the present study, we chose to study the impact of LA on p38MAPK, another member of the MAPK family. p38MAPK is also the first cell signaling molecule demonstrated to be involved in LA-induced protective effect [28], However, LA did not activate p38 MAPK in our model of myocardial I/R. This difference observed between our study and the aforementioned previous report might be due to differences in protein types and experimental protocols. Though ERK1/2, JNK1/2 and p38MAPK belong to one family, they do not necessarily work together. On the other hand, in the aforementioned study reporting the role of ERK1/2 and JNK1/2 in LA protection, the authors employed a protocol of 45 min of ischemia and 10 min of reperfusion, which is drastically different from our protocol of 30 min of ischemia and 3 h of reperfusion. This difference between protocols might explain the different results we observed regarding the role of MAPK family proteins in LA protection against MI/R.

PI3K/Akt is well documented to be protective in heart I/R injury [34]. PI3K/Akt is reported to be activated by LA and to mediate LA-induced protection against I/R in the liver [20]. We therefore hypothesize that the cardioprotection produced by LA is mediated, at least partially, by PI3K/Akt activation. In the present study, compared with the control group, the LA group showed significant increase of Akt phosphorylation, indicating its activation. More importantly, protection of LA was abolished by PI3K inhibition, indicating that LA-induced activation of Akt is pivotal for LA-mediated cardioprotection.

Among potential cytoprotective downstream targets of Akt, phosphorylation of eNOS [35] with subsequent production of NO was shown to play a key role in PI3K/Akt mediated protection, especially the anti-apoptotic effect of Akt [36]. Therefore, we examined the impact of LA pretreatment on eNOS activity and NO production. Surprisingly, even though LA did reduce cardiomyocyte apoptosis, eNOS phosphorylation was not increased in LA-treated hearts. No increase of NO was observed either. A possible explanation for this discrepancy is that LA might directly inhibit NO synthesis [37]. In the late phase of reperfusion, iNOS induction produces detrimental amounts of NO, but LA has been shown to inhibit endotoxin induced iNOS expression [21]. In the present study, LA did not inhibit iNOS induction and subsequent NO production in the late phase of reperfusion injury. This observation suggests a new mechanism by which LA exerts anti-apoptotic action during I/R injury other than modulating NO production.

Nrf2 is a key regulator of endogenous antioxidant defense. Under physiological conditions, Nrf2 is bound by its cytosolic inhibitor, Keap1, and resides in the cytoplasm before it is targeted for proteosomal degradation. During oxidative stress, Nrf2 is liberated from Keap1 and enters the nucleus, where it induces expression of genes for proteins that function as antioxidants and anti-inflammatory modulators. Phosphorylation of serine/threonine residues of Nrf2 is considered to be an important mechanism mediating Nrf2 dissociation from Keap1 [38]. Among others kinases, PI3K is shown to modulate Nrf2 dissociation from Keap1 and subsequent nuclear translocation, probably through phosphorylation of its serine/threonine residues [38], [39]. Nrf2 is shown to play an important cytoprotective role in cell injuries like I/R [40] and inflammation [41]. In the present study, compared to the I/R group, LA-treated hearts have a significantly higher level of nuclear Nrf2. This observation indicates that LA induced Nrf2 nuclear translocation in I/R myocardium. LA possesses the ability to increase Nrf2 nuclear translocation and activity in various cell culture models, supporting our data [42], [43]. Given that Nrf2 nuclear translocation can induce multiple cytoprotective gene expression, this Nrf2 nuclear translocation might mediate the cardioprotective effect of LA we observed in this study. For the first time, we also observed increased transcription and expression of the HO-1 gene in hearts treated with LA. HO-1 is preferentially inducted by Nrf2 due to the large number of APRE sequencenes which Nrf2 can bind to in its promotor [24]. In addition, HO-1 is a strong cytoprotective gene. Increased HO-1 expression is known to make hearts more resilient to I/R injury [25].Therefore, HO-1 could be an important target of Nrf2 nuclear translocation induced by LA and may mediate the cardioprotection of LA. Interestingly, PI3K inhibition abolished this increase of Nrf2 nuclear translocation and HO-1 gene expression expression, indicating that this effect is at least partially PI3K/Akt signaling pathway dependent.In future studies, we plan to use HO-1 knockout mice or HO-1 inhibitors to further determine its contribution in LA cardioprotection.

In summary, we demonstrated for the first time that LA preconditioning partially preserves heart function by reducing myocardial infarct size, cardiomyocytes apoptosis and inflammation in rat MI/R injury. The clinical relevance of our findings is supported by the fact that LA can produce protection in vivo. This protective effect is possibly dependent on PI3K/Akt activation with subsequent Nrf2 nuclear translocation and expression of HO-1. Given that Nrf2 activation can coordinately upregulate expression of several antioxidative enzymes playing important roles in combating oxidative stress, further research of the mechanism of LA preconditioning-induced protection are warranted.

## Supporting Information

Figure S1 **Dosage dependent cardioprotection of LA.** Compared to saline-treated control, 15, 25 or 50 mg/kg of LA pretreatment significantly reduced infarct size after 30 min of ischemia and 24 h of reperfusion. No difference of infarct size was observed among these 3 groups. 5 or 10 mg/kg of LA pretreatment did not reduced infarct size (a). All groups have similar AAR/LV % (b). The columns and errors bars represent means and SEM. *p<0.05 vs saline.
(TIF)

Figure S2 **Baseline heart function data.** Before ischemia, baseline heart function of rats was obtained via echocardiography. No differences of LVEF (a), LVESV (b) or LVEDV (c) was observed between groups.
(TIF)

Figure S3 **LY294002 abolished the protection of LA.** Compared to LA alone, co-treatment of LY294002 significantly elevated serum CK and LDH level (a,b), and increased infarct size (c) and cardiomyocyte apoptosis (d,e). LY294002 also reduced Akt phosphorylation (f), indicating its successful inhibition of PI3K. The columns and errors bars represent means and SEM. *p<0.05 vs I/R+LA. (LY: LY294002).
(TIF)

## References

[1] Grace PA (1994) Ischaemia-reperfusion injury. Br J Surg 81: 637–647. 8044536 10.1002/bjs.1800810504

[2] Piper HM, Garcia-Dorado D, Ovize M (1998) A fresh look at reperfusion injury. Cardiovasc Res 38: 291–300. 9709390 10.1016/s0008-6363(98)00033-9

[3] Bolli R, Becker L, Gross G, Mentzer R Jr, Balshaw D, et al (2004) Myocardial protection at a crossroads: the need for translation into clinical therapy. Circ Res 95: 125–134. 15271864 10.1161/01.RES.0000137171.97172.d7

[4] Packer L (1998) alpha-Lipoic acid: a metabolic antioxidant which regulates NF-kappa B signal transduction and protects against oxidative injury. Drug Metab Rev 30: 245–275. 9606603 10.3109/03602539808996311

[5] Goraca A, Huk-Kolega H, Piechota A, Kleniewska P, Ciejka E, et al (2011) Lipoic acid - biological activity and therapeutic potential. Pharmacol Rep 63: 849–858. 22001972 10.1016/s1734-1140(11)70600-4

[6] Coleman MD, Eason RC, Bailey CJ (2001) The therapeutic use of lipoic acid in diabetes: a current perspective. Environ Toxicol Pharmacol 10: 167–172. 21782573 10.1016/s1382-6689(01)00080-1

[7] Yan GT, Si YL, Zhang JY, Deng ZH, Xue H (2011) [The role of Leptin on neuron apoptosis in mice with cerebral ischemia/reperfusion injury]. Zhongguo Wei Zhong Bing Ji Jiu Yi Xue 23: 345–348. 21672382

[8] Bitar MS, Ayed AK, Abdel-Halim SM, Isenovic ER, Al-Mulla F (2010) Inflammation and apoptosis in aortic tissues of aged type II diabetes: amelioration with alpha-lipoic acid through phosphatidylinositol 3-kinase/Akt- dependent mechanism. Life Sci 86: 844–853. 20388520 10.1016/j.lfs.2010.03.019

[9] Richard MJ, Connell BJ, Khan BV, Saleh TM (2011) Cellular mechanisms by which lipoic acid confers protection during the early stages of cerebral ischemia: a possible role for calcium. Neurosci Res 69: 299–307. 21185885 10.1016/j.neures.2010.12.011

[10] He L, Liu B, Dai Z, Zhang HF, Zhang YS, et al (2012) Alpha lipoic acid protects heart against myocardial ischemia-reperfusion injury through a mechanism involving aldehyde dehydrogenase 2 activation. Eur J Pharmacol 678: 32–38. 22266491 10.1016/j.ejphar.2011.12.042

[11] Shaafi S, Afrooz MR, Hajipour B, Dadadshi A, Hosseinian MM, et al (2011) Anti-oxidative effect of lipoic Acid in spinal cord ischemia/reperfusion. Med Princ Pract 20: 19–22. 21160208 10.1159/000319772

[12] Pugazhenthi S, Akhov L, Selvaraj G, Wang M, Alam J (2007) Regulation of heme oxygenase-1 expression by demethoxy curcuminoids through Nrf2 by a PI3-kinase/Akt-mediated pathway in mouse beta-cells. Am J Physiol Endocrinol Metab 293: E645–655. 17535857 10.1152/ajpendo.00111.2007

[13] Ogborne RM, Rushworth SA, O’Connell MA (2005) Alpha-lipoic acid-induced heme oxygenase-1 expression is mediated by nuclear factor erythroid 2-related factor 2 and p38 mitogen-activated protein kinase in human monocytic cells. Arterioscler Thromb Vasc Biol 25: 2100–2105. 16123320 10.1161/01.ATV.0000183745.37161.6e

[14] Cheng PY, Lee YM, Shih NL, Chen YC, Yen MH (2006) Heme oxygenase-1 contributes to the cytoprotection of alpha-lipoic acid via activation of p44/42 mitogen-activated protein kinase in vascular smooth muscle cells. Free Radic Biol Med 40: 1313–1322. 16631521 10.1016/j.freeradbiomed.2005.11.024

[15] Visioli F, Smith A, Zhang W, Keaney JF Jr, Hagen T, et al (2002) Lipoic acid and vitamin C potentiate nitric oxide synthesis in human aortic endothelial cells independently of cellular glutathione status. Redox Rep 7: 223–227. 12396668 10.1179/135100002125000604

[16] Black SC, Rodger IW (1996) Methods for studying experimental myocardial ischemic and reperfusion injury. J Pharmacol Toxicol Methods 35: 179–190. 8823664 10.1016/1056-8719(96)00051-2

[17] Sun Z, Shen L, Sun X, Tong G, Sun D, et al (2011) Variation of NDRG2 and c-Myc expression in rat heart during the acute stage of ischemia/reperfusion injury. Histochem Cell Biol 135: 27–35. 21193923 10.1007/s00418-010-0776-9

[18] Tong G, Sun Z, Wei X, Gu C, Kaye AD, et al (2011) U50,488H postconditioning reduces apoptosis after myocardial ischemia and reperfusion. Life Sci 88: 31–38. 21034750 10.1016/j.lfs.2010.10.018

[19] Oh SK, Yun KH, Yoo NJ, Kim NH, Kim MS, et al (2009) Cardioprotective effects of alpha-lipoic Acid on myocardial reperfusion injury: suppression of reactive oxygen species generation and activation of mitogen-activated protein kinase. Korean Circ J 39: 359–366. 19949619 10.4070/kcj.2009.39.9.359 PMC2771824

[20] Muller C, Dunschede F, Koch E, Vollmar AM, Kiemer AK (2003) Alpha-lipoic acid preconditioning reduces ischemia-reperfusion injury of the rat liver via the PI3-kinase/Akt pathway. Am J Physiol Gastrointest Liver Physiol 285: G769–778. 12816756 10.1152/ajpgi.00009.2003

[21] Demarco VG, Scumpia PO, Bosanquet JP, Skimming JW (2004) alpha-lipoic acid inhibits endotoxin-stimulated expression of iNOS and nitric oxide independent of the heat shock response in RAW 264.7 cells. Free Radic Res 38: 675–682. 15453632 10.1080/10715760410001702503

[22] Yamada M, Kaibori M, Tanaka H, Habara K, Hijikawa T, et al (2012) alpha-lipoic acid prevents the induction of iNOS gene expression through destabilization of its mRNA in proinflammatory cytokine-stimulated hepatocytes. Dig Dis Sci 57: 943–951. 22212728 10.1007/s10620-011-2012-4

[23] Wildhirt SM, Weismueller S, Schulze C, Conrad N, Kornberg A, et al (1999) Inducible nitric oxide synthase activation after ischemia/reperfusion contributes to myocardial dysfunction and extent of infarct size in rabbits: evidence for a late phase of nitric oxide-mediated reperfusion injury. Cardiovasc Res 43: 698–711. 10690341 10.1016/s0008-6363(99)00080-2

[24] Kensler TW, Wakabayashi N, Biswal S (2007) Cell survival responses to environmental stresses via the Keap1-Nrf2-ARE pathway. Annu Rev Pharmacol Toxicol 47: 89–116. 16968214 10.1146/annurev.pharmtox.46.120604.141046

[25] Melo LG, Agrawal R, Zhang L, Rezvani M, Mangi AA, et al (2002) Gene therapy strategy for long-term myocardial protection using adeno-associated virus-mediated delivery of heme oxygenase gene. Circulation 105: 602–607. 11827926 10.1161/hc0502.103363

[26] Dudek M, Bednarski M, Bilska A, Iciek M, Sokolowska-Jezewicz M, et al (2008) The role of lipoic acid in prevention of nitroglycerin tolerance. Eur J Pharmacol 591: 203–210. 18616939 10.1016/j.ejphar.2008.06.073

[27] Ghibu S, Richard C, Vergely C, Zeller M, Cottin Y, et al (2009) Antioxidant properties of an endogenous thiol: Alpha-lipoic acid, useful in the prevention of cardiovascular diseases. J Cardiovasc Pharmacol 54: 391–398. 19998523 10.1097/fjc.0b013e3181be7554

[28] Konrad D, Somwar R, Sweeney G, Yaworsky K, Hayashi M, et al (2001) The antihyperglycemic drug alpha-lipoic acid stimulates glucose uptake via both GLUT4 translocation and GLUT4 activation: potential role of p38 mitogen-activated protein kinase in GLUT4 activation. Diabetes 50: 1464–1471. 11375349 10.2337/diabetes.50.6.1464

[29] Huang HC, Nguyen T, Pickett CB (2002) Phosphorylation of Nrf2 at Ser-40 by protein kinase C regulates antioxidant response element-mediated transcription. J Biol Chem 277: 42769–42774. 12198130 10.1074/jbc.M206911200

[30] Steffens S, Montecucco F, Mach F (2009) The inflammatory response as a target to reduce myocardial ischaemia and reperfusion injury. Thromb Haemost 102: 240–247. 19652874 10.1160/TH08-12-0837

[31] Rojo AI, Sagarra MR, Cuadrado A (2008) GSK-3beta down-regulates the transcription factor Nrf2 after oxidant damage: relevance to exposure of neuronal cells to oxidative stress. J Neurochem 105: 192–202. 18005231 10.1111/j.1471-4159.2007.05124.x

[32] Wu CC, Hsu MC, Hsieh CW, Lin JB, Lai PH, et al (2006) Upregulation of heme oxygenase-1 by Epigallocatechin-3-gallate via the phosphatidylinositol 3-kinase/Akt and ERK pathways. Life Sci 78: 2889–2897. 16378625 10.1016/j.lfs.2005.11.013

[33] Na HK, Kim EH, Jung JH, Lee HH, Hyun JW, et al (2008) (−)-Epigallocatechin gallate induces Nrf2-mediated antioxidant enzyme expression via activation of PI3K and ERK in human mammary epithelial cells. Arch Biochem Biophys 476: 171–177. 18424257 10.1016/j.abb.2008.04.003

[34] Hausenloy DJ, Yellon DM (2004) New directions for protecting the heart against ischaemia-reperfusion injury: targeting the Reperfusion Injury Salvage Kinase (RISK)-pathway. Cardiovasc Res 61: 448–460. 14962476 10.1016/j.cardiores.2003.09.024

[35] Gao F, Gao E, Yue TL, Ohlstein EH, Lopez BL, et al (2002) Nitric oxide mediates the antiapoptotic effect of insulin in myocardial ischemia-reperfusion: the roles of PI3-kinase, Akt, and endothelial nitric oxide synthase phosphorylation. Circulation 105: 1497–1502. 11914261 10.1161/01.cir.0000012529.00367.0f

[36] Chan TO, Rittenhouse SE, Tsichlis PN (1999) AKT/PKB and other D3 phosphoinositide-regulated kinases: kinase activation by phosphoinositide-dependent phosphorylation. Annu Rev Biochem 68: 965–1014. 10872470 10.1146/annurev.biochem.68.1.965

[37] Apopa PL, He X, Ma Q (2008) Phosphorylation of Nrf2 in the transcription activation domain by casein kinase 2 (CK2) is critical for the nuclear translocation and transcription activation function of Nrf2 in IMR-32 neuroblastoma cells. J Biochem Mol Toxicol 22: 63–76. 18273910 10.1002/jbt.20212

[38] Surh YJ, Kundu JK, Na HK (2008) Nrf2 as a master redox switch in turning on the cellular signaling involved in the induction of cytoprotective genes by some chemopreventive phytochemicals. Planta Med 74: 1526–1539. 18937164 10.1055/s-0028-1088302

[39] Jain AK, Jaiswal AK (2007) GSK-3beta acts upstream of Fyn kinase in regulation of nuclear export and degradation of NF-E2 related factor 2. J Biol Chem 282: 16502–16510. 17403689 10.1074/jbc.M611336200

[40] Ashrafian H, Czibik G, Bellahcene M, Aksentijevic D, Smith AC, et al (2012) Fumarate is cardioprotective via activation of the Nrf2 antioxidant pathway. Cell Metab 15: 361–371. 22405071 10.1016/j.cmet.2012.01.017 PMC3314920

[41] Kim J, Cha YN, Surh YJ (2010) A protective role of nuclear factor-erythroid 2-related factor-2 (Nrf2) in inflammatory disorders. Mutat Res 690: 12–23. 19799917 10.1016/j.mrfmmm.2009.09.007

[42] Pickering AM, Linder RA, Zhang H, Forman HJ, Davies KJ (2012) Nrf2-dependent induction of proteasome and Pa28alphabeta regulator are required for adaptation to oxidative stress. J Biol Chem 287: 10021–10031. 22308036 10.1074/jbc.M111.277145 PMC3323025

[43] Shay KP, Michels AJ, Li W, Kong AN, Hagen TM (2012) Cap-independent Nrf2 translation is part of a lipoic acid-stimulated detoxification stress response. Biochim Biophys Acta 1823: 1102–1109. 22521877 10.1016/j.bbamcr.2012.04.002 PMC4012555

