# Supplementary figures and images for: α-Lipoic Acid Reduces Infarct Size and Preserves Cardiac Function in Rat Myocardial Ischemia/Reperfusion Injury through Activation of PI3K/Akt/Nrf2 Pathway
Source: PLoS One. 2013 Mar 7;8(3):e58371. doi: 10.1371/journal.pone.0058371 (PMC3591314; doi:10.1371/journal.pone.0058371)

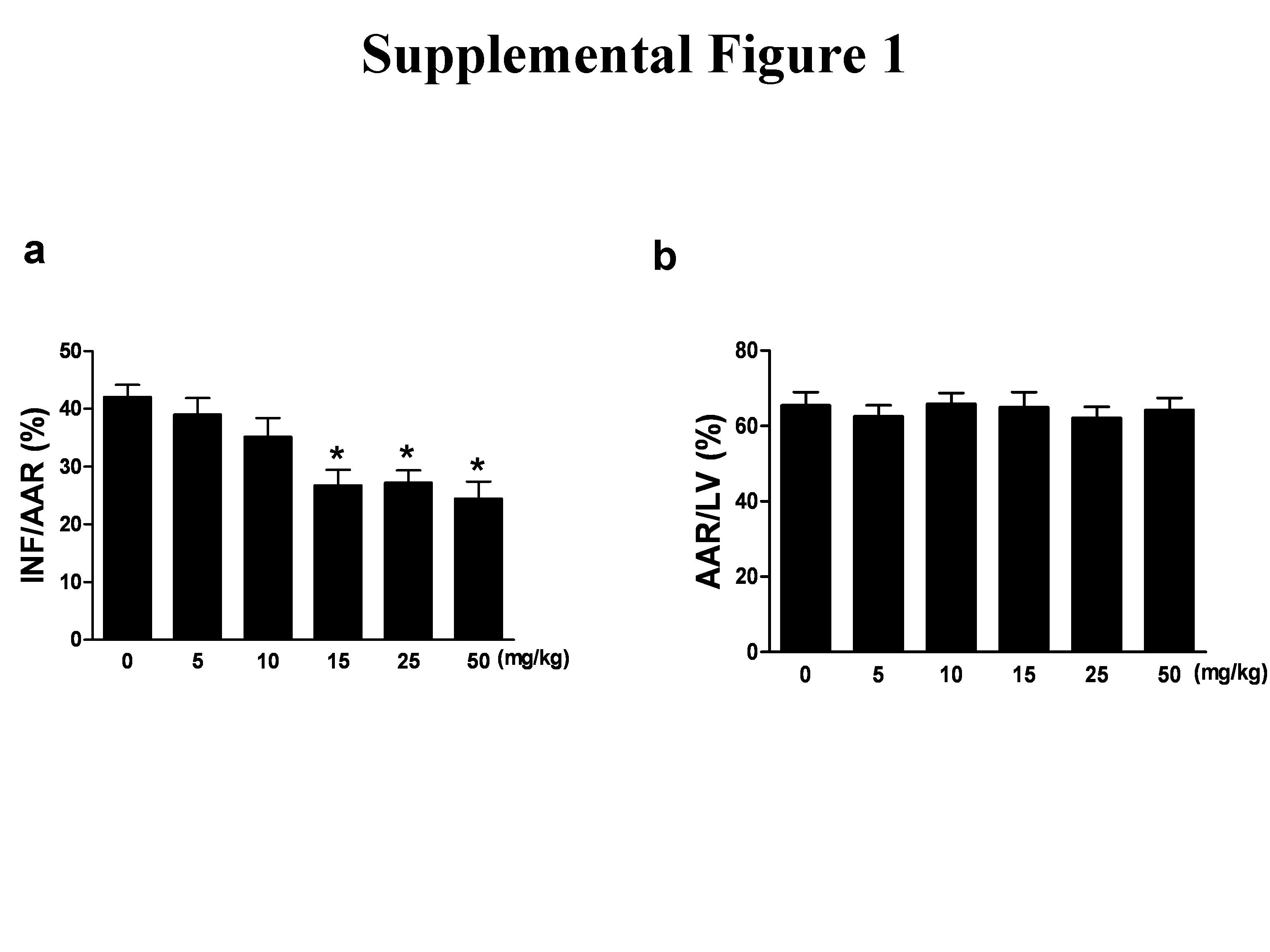

Supplement: Figure S1 — Dosage dependent cardioprotection of LA. Compared to saline-treated control, 15, 25 or 50 mg/kg of LA pretreatment significantly reduced infarct size after 30 min of ischemia and 24 h of reperfusion. No difference of infarct size was observed among these 3 groups. 5 or 10 mg/kg of LA pretreatment did not reduced infarct size (a). All groups have similar AAR/LV % (b). The columns and errors bars represent means and SEM. *p<0.05 vs saline. (TIF) [file pone.0058371.s001.tif]

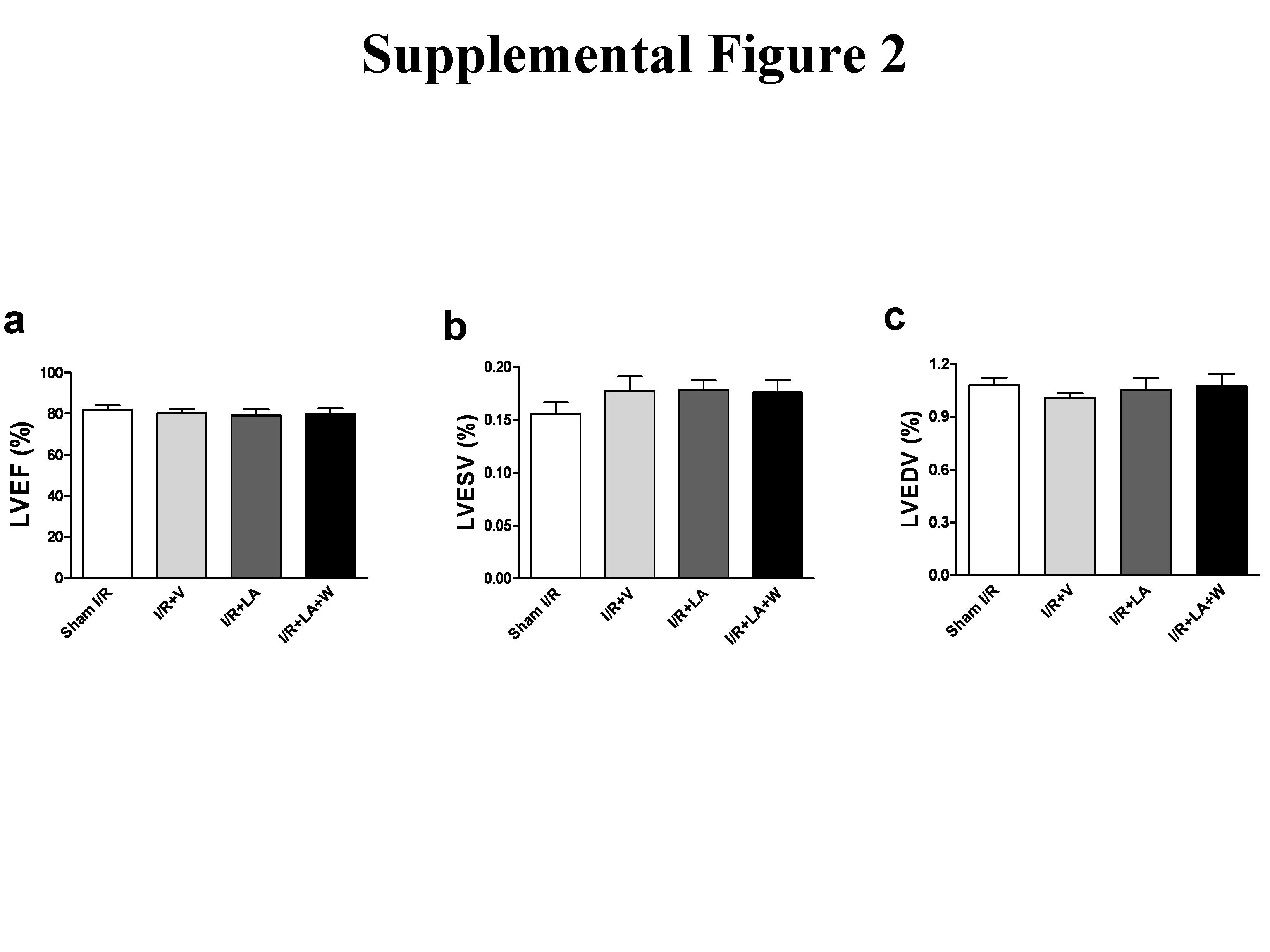

Supplement: Figure S2 — Baseline heart function data. Before ischemia, baseline heart function of rats was obtained via echocardiography. No differences of LVEF (a), LVESV (b) or LVEDV (c) was observed between groups. (TIF) [file pone.0058371.s002.tif]

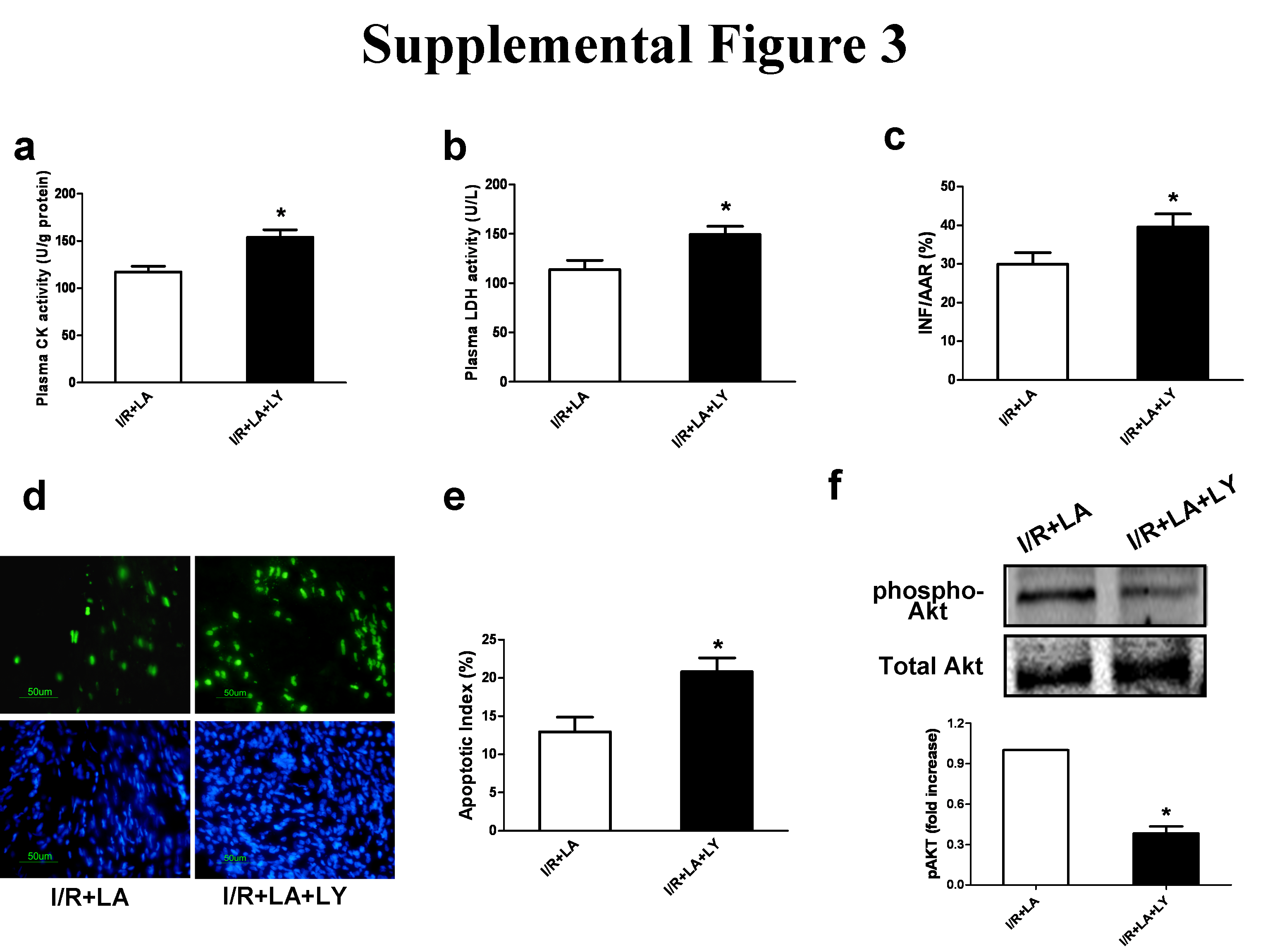

Supplement: Figure S3 — LY294002 abolished the protection of LA. Compared to LA alone, co-treatment of LY294002 significantly elevated serum CK and LDH level (a,b), and increased infarct size (c) and cardiomyocyte apoptosis (d,e). LY294002 also reduced Akt phosphorylation (f), indicating its successful inhibition of PI3K. The columns and errors bars represent means and SEM. *p<0.05 vs I/R+LA. (LY: LY294002). (TIF) [file pone.0058371.s003.tif]
